# Oseltamivir PK/PD Modeling and Simulation to Evaluate Treatment Strategies against Influenza-Pneumococcus Coinfection

**DOI:** 10.3389/fcimb.2016.00060

**Published:** 2016-06-14

**Authors:** Alessandro Boianelli, Niharika Sharma-Chawla, Dunja Bruder, Esteban A. Hernandez-Vargas

**Affiliations:** ^1^Systems Medicine of Infectious Diseases, Department of Systems Immunology and Braunschweig Integrated Centre for Infection Research, Helmholtz Centre for Infection ResearchBraunschweig, Germany; ^2^Immune Regulation, Helmholtz Centre for Infection ResearchBraunschweig, Germany; ^3^Infection Immunology, Institute of Medical Microbiology, Infection Control and Prevention, Otto-von-Guericke-UniversityMagdeburg, Germany

**Keywords:** viral infection, *S. pneumoniae* coinfection, Oseltamivir treatment, PK/PD model, microbial resistance, population modeling, viral dynamic model

## Abstract

Influenza pandemics and seasonal outbreaks have shown the potential of Influenza A virus (IAV) to enhance susceptibility to a secondary infection with the bacterial pathogen *Streptococcus pneumoniae* (Sp). The high morbidity and mortality rate revealed the poor efficacy of antiviral drugs and vaccines to fight IAV infections. Currently, the most effective treatment for IAV is by antiviral neuraminidase inhibitors. Among them, the most frequently stockpiled is Oseltamivir which reduces viral release and transmission. However, effectiveness of Oseltamivir is compromised by the emergence of resistant IAV strains and secondary bacterial infections. To date, little attention has been given to evaluate how Oseltamivir treatment strategies alter Influenza viral infection in presence of Sp coinfection and a resistant IAV strain emergence. In this paper we investigate the efficacy of current approved Oseltamivir treatment regimens using a computational approach. Our numerical results suggest that the curative regimen (75 mg) may yield 47% of antiviral efficacy and 9% of antibacterial efficacy. An increment in dose to 150 mg (pandemic regimen) may increase the antiviral efficacy to 49% and the antibacterial efficacy to 16%. The choice to decrease the intake frequency to once per day is not recommended due to a significant reduction in both antiviral and antibacterial efficacy. We also observe that the treatment duration of 10 days may not provide a clear improvement on the antiviral and antibacterial efficacy compared to 5 days. All together, our *in silico* study reveals the success and pitfalls of Oseltamivir treatment strategies within IAV-Sp coinfection and calls for testing the validity in clinical trials.

## 1. Introduction

Influenza A virus (IAV) and *Streptococcus pneumoniae* (Sp) are common causative agents of morbidity and mortality, respectively (Kilbourne, 2006; Morens et al., 2008; World Health Organization, 2009a). Over the last century four major influenza pandemics in 1918, 1957, 1968, and 2009 have had a significant impact worldwide. The Great Pandemic also known as the Spanish flu of 1918/1919 is considered as the deadliest pandemic with an estimated mortality of about 100 million around the globe (Johnson and Mueller, 2002). Interestingly, during the 1918 pandemic over 71% of the blood and sputum samples from fatal victims tested positive for Sp (Louria et al., 1959; McCullers and Rehg, 2002; McCullers, 2006, 2014), indicating a clear predisposition to lethal secondary bacterial infection in IAV preinfected patients.

Even though the mortality rate due to coinfections has decreased during the succeeding pandemics mostly because of antibiotic implementation, it still remains to be the most likely cause of death in 10–55% of the 2009 H1N1 victims. Thus, bacterial coinfection is a critical clinical outcome of viral infection and great attempts have been made to understand the pathogenesis and treatment course. The underlying mechanism for copathogenesis has been widely studied in animal models, providing evidence for a multifaceted disease affecting both lung physiology and immune responses (Shahangian et al., 2009; Small et al., 2010; Kash et al., 2011; Li et al., 2012). IAV-mediated immune aberrations such as immune cell dysfunction and apoptosis, dysregulated cytokine milieu and immunopathology in the lungs (Murray et al., 2014) have been implicated to have both immediate and long-term effects on anti-pneumococcal defense. The impact of coinfection is not limited to the bacterial outgrowth but also impairs antiviral immunity. Therefore, it is important for clinical treatment of coinfections to have a combinatorial approach to focus on all aspects of disease pathogenesis: the virus, bacteria, and host immune responses.

For prevention and treatment of acute IAV infection, antiviral drugs are an important adjunct to influenza vaccines (Goldstein and Lipsitch, 2009). The most commonly used Food and Drug Administration approved (FDA) antiviral drugs are neuraminidase inhibitors, e.g., Zanamivir, Peramivir, and Oseltamivir. The viral neuraminidase is an enzyme found on IAV surface enabling IAV virions to be released from the infected host cell. The neuraminidase inhibitors block this activity, thus interfering with viral spread and infectivity in the lungs (Moscona, 2005). *In vivo* administration of Oseltamivir is effective in controlling viral loads and immunopathology during lethal infection (McNicholl and McNicholl, 2001). In humans, the drug reduces clinical symptoms by 0.7–1.5 days when treatment is started 2 days after laboratory confirmed influenza, representing great potential if used appropriately to prevent the development of resistance (McNicholl and McNicholl, 2001). In the case of coinfections, the murine study in McCullers (2004) showed that treatment with Oseltamivir improved the survival by 75% in the coinfected group which further improved after combinatorial therapy with ampicillin. The first line of therapy following pneumococcal pneumonia is penicillin or other beta lactams, however the higher inflammatory status of the lung following coinfection with highly pathogenic virus strains may call for the use of non-lytic bacteriostatic agents such as clindamycin and azithromycin (Karlström et al., 2009). Furthermore, the anti-inflammatory and immunomodulatory action of corticosteroids used to treat many immune diseases could have a potent additive effect. In fact, the *in vivo* murine study by Trappetti et al. (2009) suggested a positive role of neuraminidase in Sp biofilm formation, thus Oseltamivir would be beneficial in preventing colonization. Following this study, the inhibiting effect of the approved anti-IAV drugs (Oseltamivir and Zanamivir) on Sp neuraminidase was confirmed by an *in vitro* kinetic study (Gut et al., 2011). Despite the existing combinatorial therapies against coinfections, the cumulative effect of neuraminidase inhibitor (Oseltamivir), the correct antibiotic and corticosteroids (Dexamethasone) is yet to be studied. With the increase in Oseltamivir use, drug resistant IAV strains may emerge bearing mutations such as H275Y in neuraminidase (Sheu et al., 2008). So far, the potentially detrimental effect of such mutant virus strains on secondary bacterial infections remains elusive.

The effectiveness of the Oseltamivir treatment depends on the dose regimen, intake frequency, time delay between infection and treatment, and treatment duration. The antiviral efficacy of neuraminidase inhibitors such as Oseltamivir, Amantadine and Peramivir has been investigated experimentally (Tanaka et al., 2015) and theoretically (Handel et al., 2007; Canini et al., 2014; Kamal et al., 2015). Recently, pharmaceutical companies have taken a strategic initiative to promote the use of modeling approaches within drug projects. The value of a model-based approach for improved efficiency and decision making during the preclinical stage of drug development has been largely advocated (Visser et al., 2013). Drug administration considers mainly two phenomena, i.e., the pharmacokinetic (PK) and pharmacodynamic (PD). The PK regards the temporal distribution of drug concentration in different organs of host body, while the PD describes the effect of a drug on the organism (Lahoz-Beneytez et al., 2015).

To the best of our knowledge, Oseltamivir treatment strategies for IAV infection in presence of Sp coinfection and a resistant IAV strain has not been investigated. In this paper, we tested the approved Oseltamivir treatment efficacy, combining a mathematical model of IAV-Sp coinfection with the PK/PD model of Oseltamivir. A possible emergence of an IAV Oseltamivir-resistant strain is also considered. Our computational results showed that Oseltamivir treatment with a dose of 150 mg, twice per day for 5 days is the minimum requirement recommended to achieve an antiviral efficacy of 49% and an antibacterial efficacy of 16%. Moreover, we found that in case of 75 mg dose administration, the intake frequency should not be lower than twice per day. A prolongation of the treatment up to 10 days with an intake frequency of twice per day, did not produce a clear benefit in terms of efficacy. This theoretical framework revealed the success and pitfalls of Oseltamivir strategies within IAV-Sp coinfection, paving the way for further refinement of therapeutic applications and clinical trials.

## 2. Materials and methods

### 2.1. PK/PD model of Oseltamivir

The PK model of Oseltamivir consists of a two compartment model (Rayner et al., 2008; Wattanagoon et al., 2009; Canini et al., 2014), one for Oseltamivir phosphate (*OP*) and one for its active metabolic compound form Oseltamivir Carboxylate (*OC*). The system of ordinary differential equations describing the concentrations of *OP* and *OC* is as follows:

(1) $$\begin{array}{l} Ġ=-k_{a}G, \end{array}$$

(2) $$\begin{array}{l} \overset{\cdot }{OP}=k_{a}G-k_{f}OP, \end{array}$$

(3) $$\begin{array}{l} \overset{\cdot }{OC}=k_{f}OP-k_{e}OC, \end{array}$$

where *G* is the depot compartment representing the *OP* dose administered, before it is adsorbed inside the blood with the adsorption rate *k*_*a*_. The parameter *k*_*f*_ is the conversion rate from *OP* to *OC* and *k*_*e*_ is the *OC* elimination rate. The initial conditions of this system are *G*(0) = *Dose*, *OP*(0) = 0, *OC*(0) = 0. As the explicit effect of *OC* is to inhibit the viral release of IAV from infected cells, we modeled in similar vein to Canini et al. (2014) the *OC* action by modifying the viral replication rate to *p* = (1 − ϵ_*S*_(*t*))*p*, where ϵ_*S*_(*t*) is the time varying drug efficacy defined as a function of *OC* concentration:

(4) $$\begin{array}{l} \epsilon_{S}\left(t\right)=\frac{OC}{EC_{50}^{S}+OC}. \end{array}$$

$EC_{50}^{S}$ is the *OC* concentration providing the 50% of drug efficacy. Cell culture assays found the values of $EC_{50}^{S}$ in the range [0.0008 − 35] μ*M* (Tamiflu (R), 2009). Simulation environments will be based on values of $EC_{50}^{S}$ equal to 0.5, 10, and 35 μ*M*.

### 2.2. IAV-pneumococcus coinfection model

**Figure 1:**
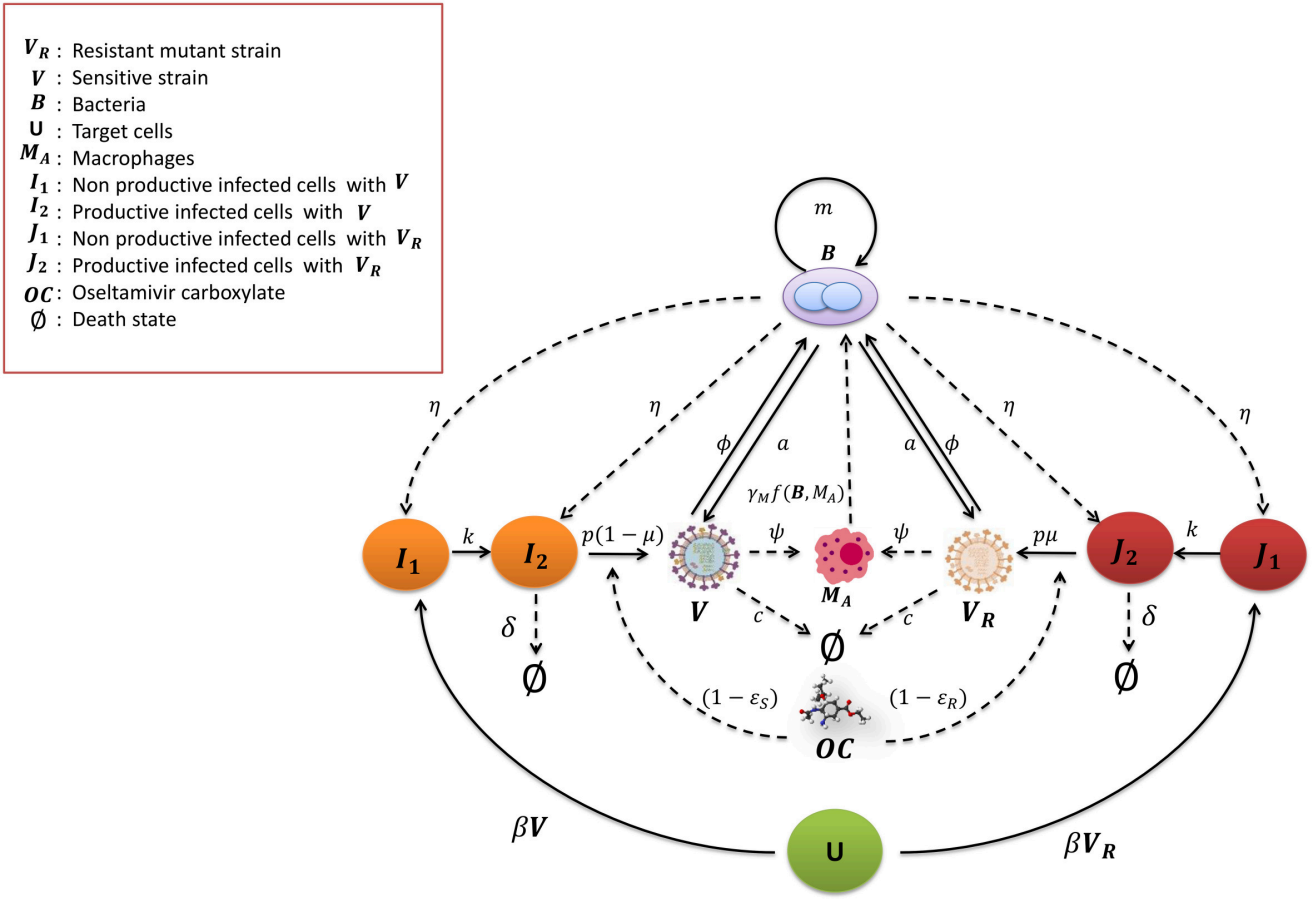
**Mathematical model for IAV-Sp coinfection with the antiviral Oseltamivir carboxylate ***OC*****. The IAV sensitive strain *V* and resistant mutant strain *V*_*R*_ (H275Y) are cleared at rate *c*. The uninfected cells become non productively infected cells *I*_1_ and *J*_1_ at the rate β by *V* and *V*_*R*_ respectively. *I*_1_ and *J*_1_ become productively infected cells at the rate *k*, that is *I*_2_ and *J*_2_ respectively. *V* and *V*_*R*_ replicate at the rate *p*(1 − μ) and μ*p* respectively. *I*_2_ and *J*_2_ are cleared at rate δ. The bacteria *B* proliferate at the rate *m* and increase the viral replication rate *a*. In turn *V* and *V*_*R*_ increase the bacterial carrying capacity with the parameter ϕ. Macrophages *M*_*A*_ phagocytose the bacteria with the rate γ_*M*_*f*(*B, M*_*A*_). The phagocytosis rate is decreased by *V* and *V*_*R*_ with the parameter ψ. Bacteria increase the toxicity rate of non productively and productively infected cells of *V* and *V*_*R*_ with the rate parameter η. The Oseltamivir carboxylate *OC* decreases the *V* and *V*_*R*_ viral replication rate *p* to *p*(1 − ϵ_*S*_(*t*)) and *p*(1 − ϵ_*R*_(*t*)) respectively.

The scheme of the mathematical model of IAV-Sp coinfection and Oseltamivir interaction is illustrated in Figure 1. The dynamic of the IAV Oseltamivir-sensitive strain *V* is described by the target cell model with the eclipse phase (Nowak and May, 2000; Baccam et al., 2006; Beauchemin and Handel, 2011; Boianelli et al., 2015). Then, this is incorporated with the mathematical model of IAV-Sp coinfection proposed by Smith et al. (2013). We denote with *U* the uninfected cells, *I*_1_ the non productively infected cells, *I*_2_ the productively infected cells. *U* is infected by *V* with infection rate β. 1∕*k* is the average time in which *I*_1_ cells become productively infected cells. δ is the clearance rate of productively infected cells *I*_2_ while *p* is the viral replication rate of *V* by *I*_2_. *c* is the viral clearance rate of *V*. We fix the initial number of uninfected cells *U*(0) at 10^7^. The initial conditions for the sensitive strain *V* and Sp *B* are in **Table 2**, while for the others model variables are set to zero. The IAV and Sp initial conditions for performing simulations are in the concentration units of TCID_50_mL^−1^ and CFUmL^−1^. The volume (mL) in the initial condition refers to the volume used (50 μL) in Smith et al. (2013) for the IAV and Sp (D39 strain) inoculum. According to the effect of *OC* on productively infected cells, the viral replication rate *p* is modified in *p*(1 − ϵ_*S*_(*t*)). We assume that an IAV Oseltamivir-resistant mutant strain (H275Y) *V*_*R*_ could emerge from the sensitive type as a consequence of Oseltamivir treatment (Sheu et al., 2008; World Health Organization, 2010; Chen et al., 2011; Dobrovolny et al., 2011; Renaud et al., 2011). The kinetic parameters of *V*_*R*_ are assumed equal to those of *V*. The emergence of *V*_*R*_ is considered to be with the probability μ. *V*_*R*_ and *V* can compete for the same target cells *U* (Govorkova et al., 2010). Then, we denote with *J*_1_ and *J*_2_ the non productively and productively infected cells respectively of *V*_*R*_, where ϵ_*R*_(*t*) is the Oseltamivir efficacy against *V*_*R*_ having the same form in (4). It has been shown that for *V*_*R*_, $EC_{50}^{R}$ is 400 times higher than those for *V* (Gubareva et al., 2001). We also explore the case where it is 200 times higher.

To investigate the synergy between IAV and Sp, Smith et al. (2013) modeled the bacteria dynamics and interaction with alveolar macrophages *M*_*A*_. The macrophages phagocytosis rate γ_*M*_*f*(*B, M*_*A*_) of free bacteria is expressed by the mathematical function $\gamma_{M}n^{2}M_{A}∕\left(n^{2}M_{A}+B^{2}\right)$, where *n* is the maximum number of bacteria phagocytosed per *M*_*A*_, γ_*M*_ is the maximum phagocytosis rate. *M*_*A*_ cells number is considered in quasi steady state, denoted as $M_{A}^{*}$. Thus, the phagocytosis rate *f*(*B, M*_*A*_) is a decreasing function of *B*. The pneumococcus growth is assumed to be logistic with rate *m* and carrying capacity *K*_*B*_. The IAV is assumed to increase the pneumococcal adherence to epithelial cells. This is translated by increasing the bacterial carrying capacity *K*_*B*_(1 + ϕ*V*) where ϕ is a proportionality constant. Moreover, another contribution of the IAV is the decreased rate of phagocytosis by *M*_*A*_. This effect is included with the saturation function ψ*V*∕(*K*_*PV*_ + *V*), where ψ is the maximal reduction of the phagocytosis rate and *K*_*PV*_ is the half saturation constant. On the other hand, the pneumococcus effects on the IAV that may cause viral rebound are unknown. One plausible hypothesis assumes that the bacterial neuraminidase supports the viral neuraminidase to enhance the viral particle release from infected cells (McCullers, 2014). This is taken into account by considering an additional term in the viral replication rate *p*(1 + *aB*^*z*^), where *z* is the nonlinearity order coefficient and *a* is the positive term of bacterial effect. The model also included the toxicity effect of *B* on *I*_1_, *J*_1_, and *I*_2_, *J*_2_ with the toxicity rate η. This model is extended including the dynamics of the resistant virus, assuming that *V*_*R*_ influences *B* in the same way of *V* and *vice versa*. The modified model is as follows:

(5) $$\begin{array}{l} \overset{\cdot }{U}=-\beta U\left(V+V_{R}\right), \end{array}$$

(6) $$\begin{array}{l} \overset{\cdot }{I_{1}}=\beta UV-kI_{1}-\eta BI_{1}, \end{array}$$

(7) $$\begin{array}{l} \overset{\cdot }{I_{2}}=kI_{1}-\delta I_{2}-\eta BI_{2} \end{array}$$

(8) $$\begin{array}{l} \overset{\cdot }{J_{1}}=\beta UV_{R}-kJ_{1}-\eta BJ_{1}, \end{array}$$

(9) $$\begin{array}{l} \overset{\cdot }{J_{2}}=kJ_{1}-\delta J_{2}-\eta BJ_{2}, \end{array}$$

(10) $$\begin{array}{l} \overset{\cdot }{V}=\left(1-\mu \right)p\left(1+aB^{z}\right)\left(1-\epsilon_{S}\left(t\right)\right)I_{2}-cV, \end{array}$$

(11) $$\begin{array}{l} \overset{\cdot }{V_{R}}=\mu p\left(1+aB^{z}\right)\left(\left(1-\epsilon_{S}\left(t\right)\right)I_{2}+\left(1-\epsilon_{R}\left(t\right)\right)J_{2}\right)-cV_{R}, \end{array}$$

(12) $$\begin{array}{l} Ḃ=mB\left(1-\frac{B}{K_{B}\left(1+\phi \left(V+V_{R}\right)\right)}\right)\\ -\gamma_{M}\frac{n^{2}M_{A}^{*}}{n^{2}M_{A}^{*}+B^{2}}B\left(1-\frac{\psi \left(V+V_{R}\right)}{V+V_{R}+K_{PV}}\right). \end{array}$$

The parameters value used for our population approach are in Table 1. These values represent the median value estimated in Canini et al. (2014), Wattanagoon et al. (2009) and Smith et al. (2013). More specifically, IAV-Sp model parameters were estimated from adult mice in Smith et al. (2013). It should be noted that kinetics and time scales of viral titer as well as immune parameters estimated from murine data can offer a reasonable approximation of IAV-Sp dynamics in humans (Small et al., 2010; Beauchemin and Handel, 2011). The parameter μ was estimated from human studies in Hayden (2001), as well as the Oseltamivir PK/PD model was inferred for human adults (Wattanagoon et al., 2009).

**Table 1:** **IAV-Sp and PK/PD Oseltamivir model parameters with ranges used for the population approach**.

**Parameter**	**Definition**	**Median (Range)^a^**	**Unit**	**References**
**IAV-Sp MODEL PARAMETERS**				
β	Virus infectivity	2.8 (1.96 3.64) × 10^−6^	TCID_50_mL^−1^	Smith et al., 2013
*k*	Eclipse phase	4.0 (2.8 5.2)	day^−1^	Smith et al., 2013
δ	Productive cell clearance rate	0.89 (0.62 1.16)	day^−1^	Smith et al., 2013
*p*	Viral replication rate	25.1 (17.7 32.89)	TCID_50_mL^−1^day^−1^	Smith et al., 2013
*c*	Viral clearance rate	28.4 (19.88 36.92)	day^−1^	Smith et al., 2013
η	Toxicity of infected cell rate	5.2 (3.64 6.76) × 10^−10^	CFU mL^−1^	Smith et al., 2013
μ	Resistant virus appearance rate	2 (1.4 2.6) × 10^−6^	adim	Hayden, 2001
ϕ	Increase in carrying capacity	1.2 (0.84 1.56) × 10^−8^	TCID_50_mL^−1^	Smith et al., 2013
ψ	Decrease in phagocytosis rate	0.87 (0.61 1.13)	adim	Smith et al., 2013
*a*	Positive feedback rate	1.2 (0.84 1.56) × 10^−3^	CFU mL^−*z*^	Smith et al., 2013
*m*	Bacterial growth rate	27 (19 35)	day^−1^	Smith et al., 2013
*K*_*B*_	Pneumococcus carrying capacity	2.3 (1.61 2.99) × 10^8^	CFUmL^−1^	Smith et al., 2013
*K*_*PV*_	Half saturation constant	1.8 (1.26 2.34) × 10^3^	TCID_50_mL^−1^	Smith et al., 2013
γ_*M*_	Macrophages phagocytosis rate	1.35 (0.95 1.75) × 10^−4^	cell^−1^day^−1^	Smith et al., 2013
*n*	Maximum bacteria number for *M*_*A*_	5.0 (3.5 6.5)	CFUmL^−1^ cell^−1^	Smith et al., 2013
*z*	Non linear coefficient	0.5 (0.35 0.65)	adim	Smith et al., 2013
**OSELTAMIVIR PK/PD PARAMETERS**				
*k*_*a*_	OP adsorption rate	1.01 (0.7 1.31)	h^−1^	Wattanagoon et al., 2009
*k*_*f*_	OP conversion rate in OC	0.684 (0.48 0.88)	h^−1^	Wattanagoon et al., 2009
*k*_*e*_	OC clearance rate	0.136 (0.09 0.177)	h^−1^	Wattanagoon et al., 2009

a *Parameter ranges used for the population approach. The values are computed with ± 30% of variation from the median values*.

*The volume (mL) in model parameters was related to the total volume used (50 μL) in Smith et al. (2013) for the IAV and Sp (D39 strain) inoculum*.

#### 2.2.1. Drug regimen evaluation

The approved regimens stated by the guidelines for Oseltamivir administration in human adults (World Health Organization, 2009b; Canini et al., 2014) are: 75 mg twice per day for 5 days (curative regimen), 150 mg twice per day for 5 days (recommended regimen for pandemic influenza). These regimens are shown in Table 2 as a benchmark for the treatment evaluation. Oseltamivir regimens were evaluated with the antiviral efficacy index defined in Canini et al. (2014) as:

(13) $$\begin{array}{l} VEFF=1-\frac{AUCV_{T}+AUCR_{T}}{AUCV+AUCR}, \end{array}$$

where AUCV_*T*_ and AUCR_*T*_ are the area under the curve of *V* and *V*_*R*_ in presence of treatment, while AUCV and AUCR are the area under the curve without treatment. We also computed the antibacterial efficacy of the Oseltamivir treatment:

(14) $$\begin{array}{l} BEFF=1-\frac{AUCB_{T}}{AUCB}, \end{array}$$

where AUCB_*T*_ and AUCB are the area under the curve of the bacterial time course with and without treatment.

**Table 2:** **Simulation settings and approved Oseltamivir treatment regimens**.

**Variable**	**Range**	**Units**
Therapy initiation time	[2 3 4]	days
Time of pneumococcus coinfection after influenza infection	[4 5 6 7]	days
Intial viral load/titer	[2 100]	TCID_50_mL^−1^
Initial pneumococcal (D39 strain) load	[20 600]	CFU mL^−1^
**APPROVED REGIMENS (World Health Organization, 2009b)**		
**Dose**	**Intake frequency**	**Treatment duration**
75 mg (curative)	Twice per day	5 days
150 mg (pandemic)	Twice per day	5 days

*The volume (mL) was related to that used (50 μL) in Smith et al. (2013) for the IAV and Sp (D39 strain) inoculum*.

#### 2.2.2. Population approach

In order to take into account the individual heterogeinity observed *in vivo* (Canini and Carrat, 2011), we performed 10,000 simulations by sampling from a uniform distribution centered in the estimated values of Table 1 with a variation of ±30%. Model parameter ranges are showed in Table 1. We computed the antiviral and antibacterial efficacy defined in Equations (13)–(14) of the curative regimen with 75, 150, 300, and 450 mg, twice per day for 5 days. Moreover, a different intake frequency of once per day for 5 days with dosage of 75 mg was explored. A different treatment duration of 10 days with 75 mg and intake frequency of twice per day was also investigated. In order to mimic a realistic scenario, we assumed a random sampling for the starting time of drug treatment, time of coinfection and initial values of viral and bacterial titers. In fact, the amount of viral and bacterial burden is unknown when an individual is infected by IAV and Sp. Moreover, it is also unknown after how many days post the infection time the antiviral treatment is started. This is because the time of infection is not known. In the same way, the time of coinfection is typically unknown in naturally acquired Sp coinfection. The ranges of experimental values are presented in Table 2. For the correct viral dynamics simulation, we imposed the viral titer *V* to be constant when it crosses lower values than the threshold of 2.8 × 10^−7^ TCID_50_mL^−1^. The minimum therapy initiation time was considered starting at 2 days post infection, because at this time symptoms are clearly visible (Aoki et al., 2003; Louie et al., 2012; Muthuri et al., 2012).

#### 2.2.3. Statistical analysis

We performed the one way ANOVA statistical significance test and then Bonferroni test on the 10,000 stochastic simulations. The statistical significance difference for antiviral and antibacterial distributions between the 75 mg dose (curative regimen) and 150 (pandemic regimen), 300, and 450 mg were computed. We compared also the curative regimen intake frequency of twice per day with intake frequency of once per day. Moreover, we investigated the statistical significance between treatment duration of 5 and 10 days. The comparison is done for $EC_{50}^{S}$ values of 0.5, 10, and 35 μ*M*.

#### 2.2.4. Sensitivity analysis

To analyse to which extent each parameter affected the model outputs, we simulated the viral and bacterial dynamics by changing the parameters in Table 1 once per time of 10, 30 and 50% and keeping the others fixed (see Supplementary Figures S4, S5).

## 3. Results

### 3.1. Influence of Oseltamivir dose

**Figure 2:**
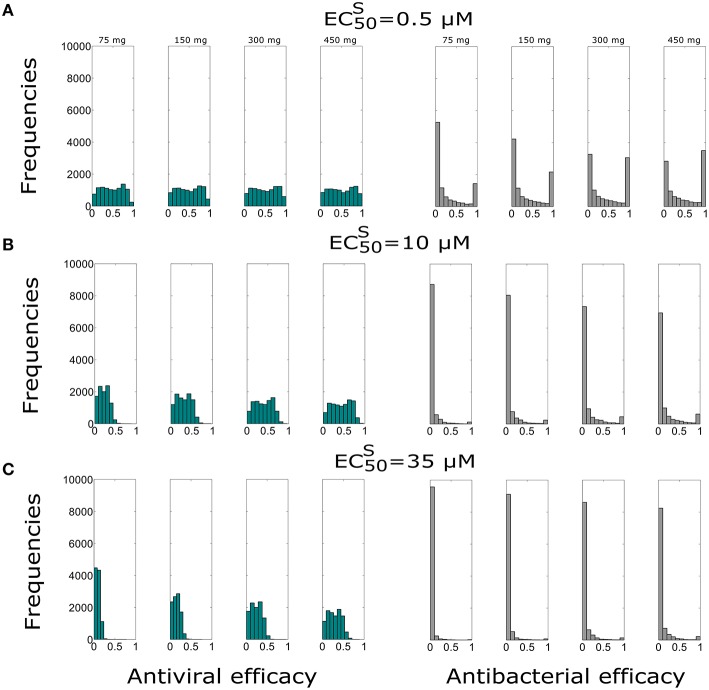
**Histograms of the antiviral (green) and antibacterial efficacy (grey) for different Oseltamivir dose of 75, 150, 300, and 450 mg, with intake frequency of twice per day and a duration of 5 days for (A) $EC_{50}^{S}=0.5$ μ***M***, (B) $EC_{50}^{S}=10$ μ*M*, and (C) $EC_{50}^{S}=35$ μ***M*****. The x-axis represents the Oseltamivir antiviral/antibacterial efficacy against IAV ranging from 0 (0%) to 1 (100%).

In this section we evaluated the antiviral and antibacterial efficacy for Oseltamivir dose of 75, 150, 300, 450 mg, twice per day for a treatment duration of 5 days. Figure 2 displays histograms obtained for different doses and $EC_{50}^{S}$. The histograms represent the distribution of antiviral and antibacterial efficacy values computed from 10,000 samples. Tables 3 and 4 show the median values of antiviral and antibacterial efficacy for different doses and $EC_{50}^{S}$ values. Interestingly, the antibacterial efficacy histograms in Figure 2 presented a bimodal distribution for both different doses and $EC_{50}^{S}$ values. The antibacterial *dichotomy* may be a result of two factors. The first could be attributed to the bacterial growth rate (*m*) and macropaghes phagocytosis rate (γ_*M*_), greatly affecting Sp dynamics. The second refers to IAV parameters responsible for macrophages phagocytosis decrease (*K*_*pv*_) and bacterial carrying capacity increase (ϕ) that influence the pneumococcal time course as well (Supplementary Figure S5). Then, combinations of these viral and bacterial parameters can promote alternatively bacterial colonization or bacterial clearance. This implies that in favorable conditions, Oseltamivir treatment strategies may be able to inhibit viral dynamics (high antiviral efficacy) and in turn modulate bacterial growth (high antibacterial efficacy). Otherwise, in worst scenarios, Oseltamivir treatment strategies may fail to control bacterial dynamics.

**Table 3:** **Antiviral efficacy median values for different $EC_{50}^{S}$ and different dose regimens with intake frequency of twice per day and treatment duration of 5 days**.

**Dose (mg)**	**$EC_{50}^{S}$ (μM)**		
	**0.5**	**10**	**35**
75	0.47^*^	0.22^*^	0.087^*^
150	0.49^*^	0.31^*^	0.153^*^
300	0.49	0.40^*^	0.237^*^
450	0.49	0.45^*^	0.289^*^

* *Statistically significant*.

*For $EC_{50}^{S}=0.5$ μM, statistically significant difference was observed between the antiviral efficacy distributions for the dose of 75 and 150 mg (P < 0.05), while significant differences were found (P < 0.05) between all the dose for $EC_{50}^{S}=10$, 35 μM*.

**Table 4:** **Antibacterial efficacy median values for different $\text{E}{\text{C}}_{50}^{S}$ and different dose regimens with intake frequency of twice per day for a duration of 5 days**.

**Dose (mg)**	**EC**^*****S*****^_**50**_ **(μM)**		
	**0.5**	**10**	**35**
75	0.09^*^	0.010^*^	0.003^*^
150	0.16^*^	0.017^*^	0.006^*^
300	0.31^*^	0.030^*^	0.010^*^
450	0.41^*^	0.036^*^	0.015^*^

* *Statistically significant*.

*For $EC_{50}^{S}=0.5$, 10, 35 μM, the differences between the antibacterial efficacy for all doses of 75, 150, 300, and 450 mg were statistically significant (P < 0.05)*.

For the best scenario where Oseltamivir treatment is effective ($EC_{50}^{S}=0.5$ μ*M*), the first set of 10,000 simulations revealed that the distribution of antiviral and antibacterial efficacy were significantly different by changing the dose from 75 to 150 mg. In fact, for the curative regimen of 75 mg, the antiviral efficacy median value was 47%, remaining quite stable for higher values of dose (49%). The same pattern was conserved for higher values of $EC_{50}^{S}$, where for the highest dosage tested (450 mg), the median antiviral efficacy values were 45 and 28.9%, while for the curative regimen were 22 and 8.7% respectively. Concurrently, in Table 4, the antibacterial efficacy for the curative regimen with $EC_{50}^{S}=0.5$ μ*M* presented a value of 9% that further decreased to 0.3% for $EC_{50}^{S}=35$ μ*M*. Similarly, for 450 mg, the antibacterial efficacy dropped from 41 to 1.5%.

We also investigated the sensitivity of the Oseltamivir antiviral and antibacterial efficacy with respect to the $EC_{50}^{R}$ values. The previous results were with $EC_{50}^{R}=400\times EC_{50}^{S}$. Thus, we tested the same treatment regimens of 75, 150, 300, and 450 mg with intake frequency of twice per day, for 5 days where $EC_{50}^{R}=200\times EC_{50}^{S}$. We computed the antiviral and antibacterial efficacy for the previous existing three different values of $EC_{50}^{S}$ (see Supplementary Figure S1). Histograms of antiviral and antibacterial efficacy presented the same properties observed when $EC_{50}^{R}=400\times EC_{50}^{S}$. More specifically, in agreement with the previous case with $EC_{50}^{R}=400\times EC_{50}^{S}$, a statistically significant difference was observed between 75 and 150 mg for the lowest value of $EC_{50}^{S}$. We noted similar median values of the antiviral efficacy for different doses and $EC_{50}^{S}$ with respect to the case where $EC_{50}^{R}=400\times EC_{50}^{S}$. In the same way, the antibacterial efficacy for 200 and 400 times the value of $EC_{50}^{S}$ also presented consistent values (see Supplementary Tables S1, S2).

### 3.2. Role of Oseltamivir intake frequency

**Figure 3:**
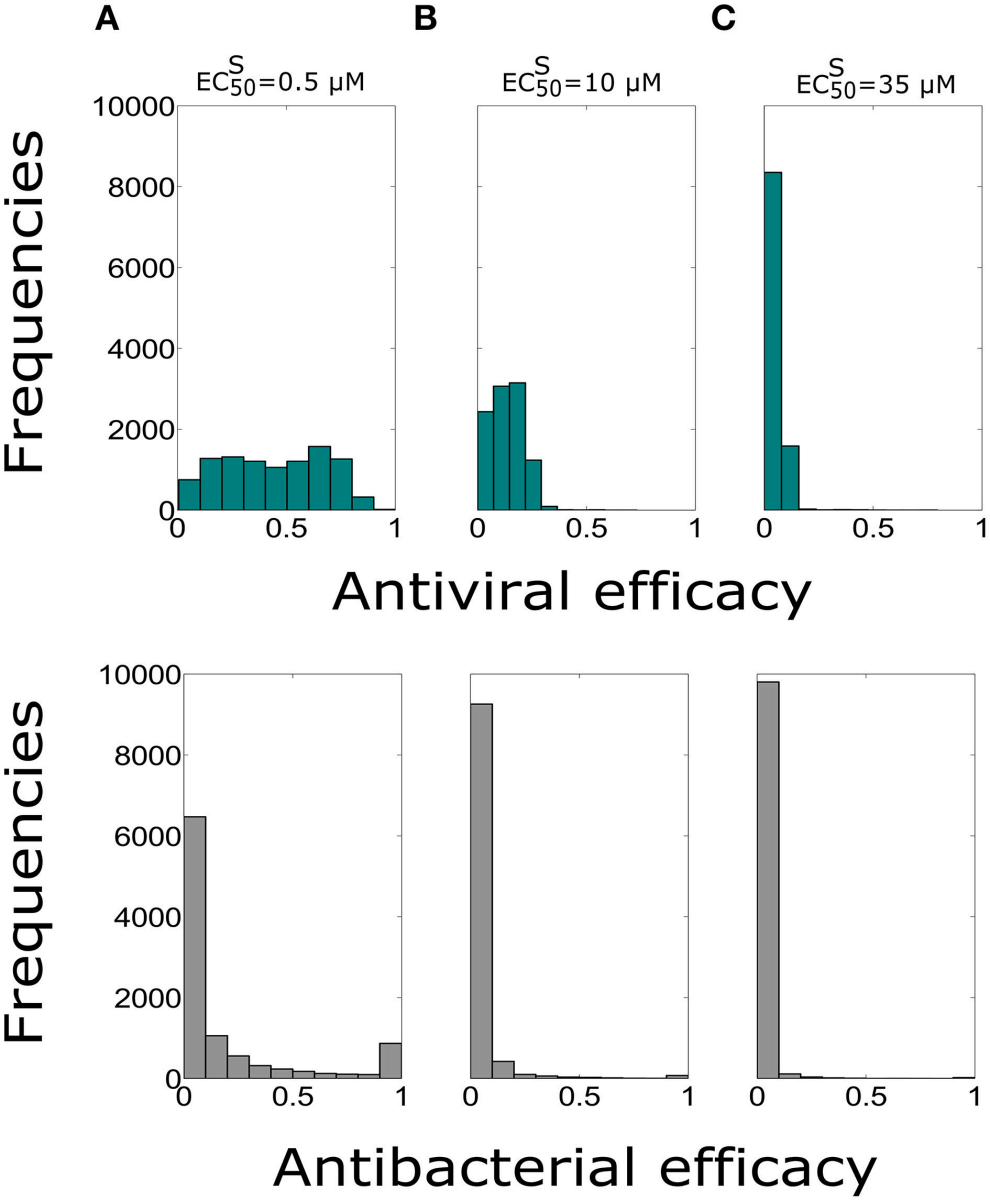
**Histograms of the antiviral (green) and antibacterial (grey) efficacy for the dose of 75 mg and intake frequency of once per day, with the duration of 5 days for (A) $EC_{50}^{S}=0.5$ μ***M***, (B) $EC_{50}^{S}=10$ μ***M***, (C) $EC_{50}^{S}=35$ μ***M*****. The x-axis represents the Oseltamivir antiviral/antibacterial efficacy against IAV/Sp ranging from 0 (0%) to 1 (100%).

In order to investigate the effect of the intake frequency treatment on the coinfection course dynamics, we simulated in another set of 10,000 simulations, the administration of 75 mg dose with intake frequency of once per day, for 5 days. These treatment regimens are explored for the same values of $EC_{50}^{S}$ considered previously and with the value of $EC_{50}^{R}=400\times EC_{50}^{S}$. The antiviral and antibacterial efficacy values are reported in Table 5. Histograms obtained from 10,000 simulations are shown in Figure 3.

**Table 5:** **Comparison of antiviral and antibacterial efficacy median values for different $\text{E}{\text{C}}_{50}^{S}$ values with intake frequency of once and twice per day and treatment duration of 5 days**.

**Intake frequency**	**EC**^*****S*****^_**50**_ **(μM)**		
	**0.5**	**10**	**35**
**ANTIVIRAL EFFICACY**			
Twice per day	0.47^*^	0.22^*^	0.09^*^
Once per day	0.43^*^	0.135^*^	0.04^*^
**ANTIBACTERIAL EFFICACY**			
Twice per day	0.09^*^	0.010^*^	0.005^*^
One per day	0.04^*^	0.005^*^	0.002^*^

* *Statistically significant*.

*Statistical significance difference of antiviral/antibacterial efficacy distributions (P < 0.05) was obtained between different intake frequency of once and twice per day*.

Both antiviral and antibacterial histogram values significantly decreased when the intake frequency was once per day or for higher $EC_{50}^{S}$. The bimodal distribution was conserved for the antibacterial histograms due to reasons stated previously. The distributions of antiviral and antibacterial efficacy presented statistically significant difference (*P* < 0.05) for both different values of intake frequency and $EC_{50}^{S}$. Notably, the values in Table 5 with intake frequency of once per day were lower compared to the median values of the antiviral efficacy of the curative regimens for different values of $EC_{50}^{S}$. Therefore, the antibacterial efficacy medians with intake frequency of once per day showed approximately half values with respect to those with intake frequency of twice per day. These results stressed the importance of intake frequency to determine the clearance of the IAV-Sp coinfection. Furthermore, the case where $EC_{50}^{R}=200\times EC_{50}^{S}$ (Supplementary Figure S2) was also investigated, noting that in this regimen both the antiviral and antibacterial efficacy medians possessed similar ranges compared with those obtained when $EC_{50}^{R}=400\times EC_{50}^{S}$ (see Supplementary Table S3).

### 3.3. Effect of treatment duration

**Figure 4:**
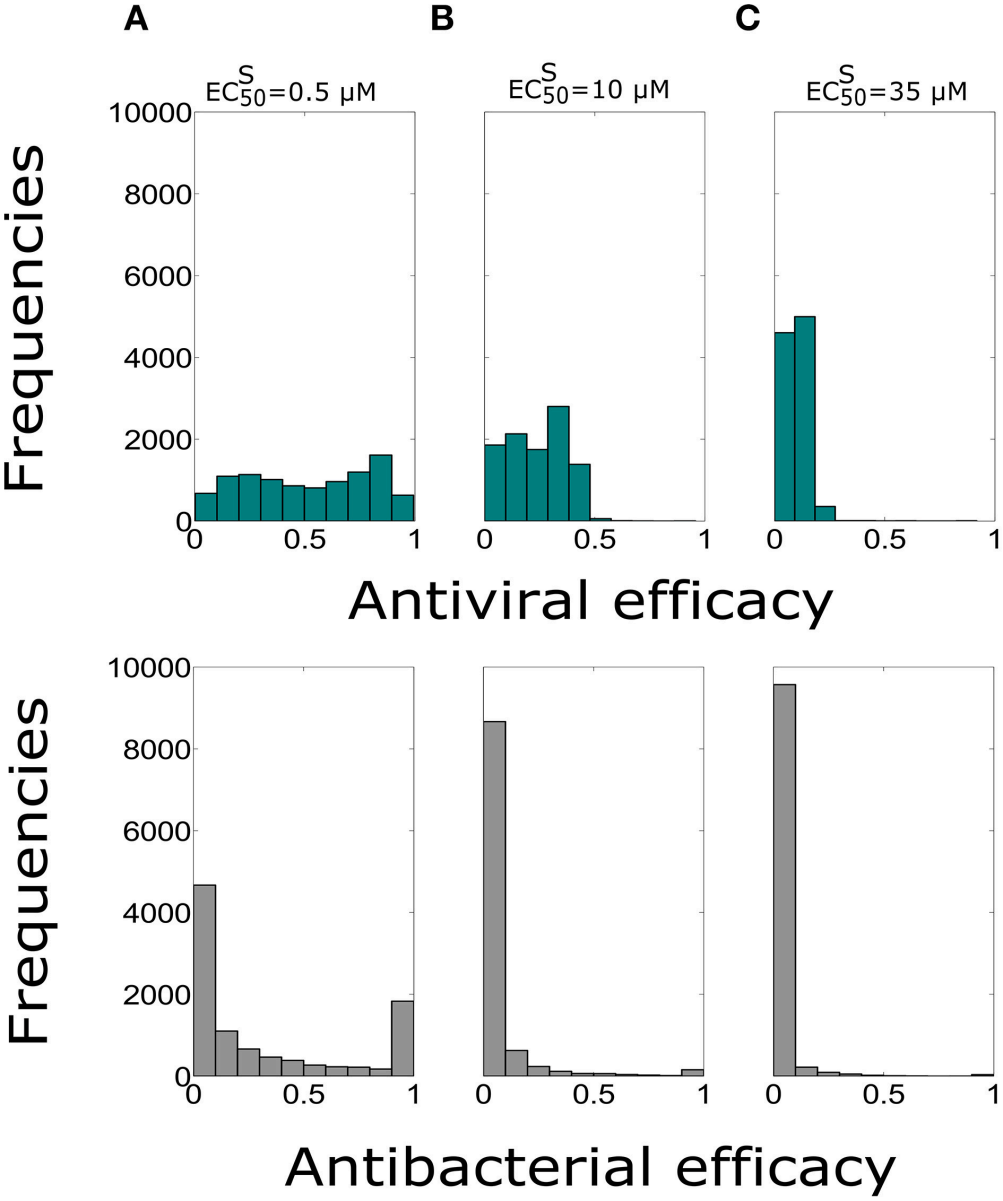
**Histograms of the antiviral (green) and antibacterial (grey) efficacy for the dose of 75 mg and intake frequency of twice per day with the duration of 10 days for (A) $EC_{50}^{S}=0.5$, (B) $EC_{50}^{S}=10$ μ***M***, (C) $EC_{50}^{S}=35$ μ***M*****. The x-axis represents the Oseltamivir antiviral/antibacterial efficacy against IAV/Sp ranging from 0 (0%) to 1 (100%).

In order to test the influence of the treatment duration on the Oseltamivir efficacy against coinfection dynamics, we assumed the possibility of treatment duration of 10 days with dose of 75 mg and intake frequency of twice per day. The median values of antiviral and antibacterial efficacy are in Table 6. The histograms showing the antiviral and antibacterial efficacy distributions obtained from 10,000 simulations for different $EC_{50}^{S}$ values are presented in Figure 4.

**Table 6:** **Comparison of antiviral and antibacterial efficacy medians for different $\text{E}{\text{C}}_{50}^{S}$ values and intake frequency of twice per day with the treatment duration of 5 and 10 days**.

**Treatment duration**	**$EC_{50}^{S}$ (μM)**		
	**0.5**	**10**	**35**
**ANTIVIRAL EFFICACY**			
5 days	0.47	0.22	0.09
10 days	0.52	0.24	0.10
**ANTIBACTERIAL EFFICACY**			
5 days	0.08	0.01	0.005
10 days	0.12	0.01	0.003

*For the antiviral/antibacterial efficacy, statistical significance difference was not found (P>0.05) for both treatment durations and different $EC_{50}^{S}$ values*.

Antiviral efficacy distributions for treatment duration of 5 and 10 days show similar median values for all of $EC_{50}^{S}$ (no statistical significance differences are noted, *P* > 0.05). Moreover, the antibacterial efficacy with treatment durations of 5 and 10 days confirmed the same pattern, in particular for $EC_{50}^{S}=10$ μ*M*. We also investigated the same treatment regimens using the value of $EC_{50}^{R}=200\times EC_{50}^{S}$ (see Supplementary Figure S3). In this setting also the antiviral efficacy distributions with treatment durations for 5 and 10 days were not statistically significant. We noted similar median values for the antiviral and antibacterial efficacy when $EC_{50}^{R}=200\times EC_{50}^{S}$ (see Supplementary Table S4), for all the values of $EC_{50}^{S}$ considered. Overall analysis suggested that the resistant mutant strain behavior may not really altered the efficacy of the Oseltamivir against IAV-Sp coinfection.

## 4. Discussion

In the last decades many mathematical models have been developed describing the IAV infection dynamics in different hosts (Baccam et al., 2006; Tridane and Kuang, 2010; Hernandez-Vargas and Meyer-Hermann, 2012; Smith et al., 2013; Canini and Perelson, 2014; Boianelli et al., 2015), and in presence of treatment (Beauchemin and Handel, 2011; Canini and Perelson, 2014; Canini et al., 2014; Boianelli et al., 2015). However, the history of IAV pandemics have highlighted the role of the secondary bacterial infection in the increased morbidity and mortality. To date, the only mathematical model describing the IAV-pneumococcus coinfection was developed by Smith et al. (2013).

In this paper, we extend the coinfection model from Smith et al. (2013), by adding the pharmacokinetic and pharmacodynamic effects of Oseltamivir and taking into account a possible emergence of resistant mutant strain (H275Y) induced by Oseltamivir treatment. In our model, we simulate the intra-subject variability of influenza infection and also a time dependent Oseltamivir drug efficacy. We test the capability of the current approved Oseltamivir treatment regimens to achieve antiviral and antibacterial efficacy in a stochastic environment. Here, we simulate a more realistic scenario for coinfection and Oseltamivir treatment strategies. For example, we assume a random time of treatment as we do not know the delay between viral infection and treatment initiation. Secondly, we consider the time of coinfection randomly, because the time of secondary Sp infection is unknown. Moreover, in real life infection the exact amount of the viral and bacterial burden is usually unknown as well. The possibility of different intake frequencies and treatment duration according to the approved treatment regimens is explored.

Our results show that the curative regimen (75 mg for 5 days, twice per day) may offer the 47% of antiviral efficacy and 9% of antibacterial efficacy only in the case where the Oseltamivir is effective ($EC_{50}^{S}=0.5$ μ*M*) against IAV. Increasing the dose from 75 to 150 mg with the same value of $EC_{50}^{S}$ results in a statistically significant gain in terms of antiviral (49%) and antibacterial efficacy (16%). Then, for the case of IAV-Sp coinfection, the pandemic regimen could be recommended. Moreover, increasing the dose may not represent a reasonable gain of antiviral and antibacterial efficacy. However, in the case of the lowest efficiency of Oseltamivir ($EC_{50}^{S}=35$ μ*M*), a significant increase in antiviral and antibacterial efficacy is obtained with a dose of 450 mg. With this dose, twice per day, for 5 days, antiviral and antibacterial efficacy display 28.9 and 1.5% median values, respectively. In the same range of treatment strategies for the value of $EC_{50}^{R}=200\times EC_{50}^{S}$ the antiviral and antibacterial efficacy presented no significant differences compared to the case where $EC_{50}^{R}=400\times EC_{50}$. Moreover, reducing the intake frequency from twice to once per day with a dose of 75 mg could determine a significant reduction in the antiviral and antibacterial efficacy for the ranges of $EC_{50}^{S}$ explored. In particular, from the best scenario ($EC_{50}^{S}=0.5$ μ*M*), the antiviral efficacy reduces from 47 to 43% and the antibacterial efficacy from 9 to 4%. This reduction is more pronounced in the worst case ($EC_{50}^{S}=35$ μ*M*), where both antiviral and antibacterial efficacy reduce approximately to the half of those values presented for a dosage of twice per day.

Against intuition, when the treatment duration is prolonged to 10 days with dose of 75 mg, this does not significantly increase the antiviral and antibacterial efficacy for all the values of $EC_{50}^{S}$. Concerning the antiviral efficacy, this result can be mainly attributed to the positive feedback of the bacterial secondary infection on IAV dynamics and in turn on the viral area under the curve. On the other hand, the antibacterial efficacy is also influenced, since the viral dynamics can modulate the bacterial growth via macrophages deactivation and can increase bacterial carrying capacity. The latter statement is also one of the factors that could lead to the bimodal distribution of the antibacterial efficacy histograms observed in all the treatment strategies. Importantly, from our computational study, the pharmacokinetic parameter $EC_{50}^{S}$ directly influences the outcome of the Oseltamivir drug on IAV-Sp coinfection for all the tested treatment regimens. On the contrary, it turns out that the sensitivity of the antiviral and antibacterial efficacy to the $EC_{50}^{R}$ parameter is low. This implies to presume that the resistant mutant strain does not really affect the antiviral and antibacterial efficacy. This is in agreement with *in silico* results obtained in Canini et al. (2014) where the authors evaluated the impact of Oseltamivir treatment strategies in the presence of the emerging resistant strain. In fact, for the treatment strategies considered in our work, the authors observed similar values of the antiviral efficacy (Treanor et al., 2000) when treatment is initiated at day 2 post infection. Therefore, our results of antibacterial efficacy (9%) obtained with curative regimen are lower than the experimental work of McCullers (2004), reporting an antibacterial efficacy value of 25% for murine data.

However, there are limitations in our simulation studies. Concerning the applied model (1)–(12), we did not consider the role of the immune response to clear the influenza virus. In fact, our investigations cannot be applied with hosts shedding preexisting immunity. Future studies should consider different models for the viral infection including the dynamics of the immune response such as CD8+T cells (Hancioglu et al., 2007; Lee et al., 2009; Miao et al., 2010; Tridane and Kuang, 2010; Dobrovolny et al., 2013), Interferon type I (Canini and Carrat, 2011; Pawelek et al., 2012; Hernandez-Vargas et al., 2014) and Natural killer cells (Canini and Carrat, 2011). In addition, the PK/PD dynamics have been estimated only for adults (Wattanagoon et al., 2009). This implies that for other groups such as children and seniors, our computational study should be tested with appropriate PK/PD parameter estimates. In fact for elderly, PK/PD parameters, e.g., apparent volume of distribution (prolongation of elimination half-life) can have important changes due to age modification in organ physiology (Mangoni and Jackson, 2004).

In summary, we find that the actual recommended regimens for Oseltamivir, i.e., curative and pandemic regimens may not completely able to control the colonization of a secondary bacterial coinfection. Higher doses, such as 150 and 300 mg, are recommended. Nevertheless, even this treatment regimen may not control coinfection in case of low Oseltamivir effectiveness. Moreover, our computational study suggests clear disadvantages of reducing the intake frequency below twice per day for a treatment duration of 5 and 10 days. Future clinical studies are needed to verify our results towards improved therapeutic treatments to fight coinfections (Dunning et al., 2014).

## Author contributions

AB and EH designed the computational study and revised the manuscript. AB performed the simulations. AB, EH, NS, and DB discussed and wrote the manuscript.

### Conflict of interest statement

The authors declare that the research was conducted in the absence of any commercial or financial relationships that could be construed as a potential conflict of interest.
