# Supplementary material for: Oseltamivir PK/PD Modeling and Simulation to Evaluate Treatment Strategies against Influenza-Pneumococcus Coinfection
Source: Front Cell Infect Microbiol. 2016 Jun 14;6:60. doi: 10.3389/fcimb.2016.00060 (PMC4906052; doi:10.3389/fcimb.2016.00060)
Supplement: Supplementary file 1 [file Presentation1.PDF]

Supplementary Material: Oseltamivir PK/PD modelling and simulation to evaluate treatment strategies against Influenza Virus-Pneumococcus coinfection

Alessandro Boianelli, Niharika Sharma-Chawla, Dunja Bruder, Esteban A. Herandez-Vargas*

*Correspondence:
Esteban A. Hernandez-Vargas
Esteban.Vargas@helmholtz-hzi.de

1 SUPPLEMENTARY TABLES AND FIGURES

Table S1. Comparison of antiviral efficacy median values for different drug efficacy EC_{50}^S and different dose regimens with intake frequency of twice per day with treatment duration of 5 days. We indicate the statistical significance ($P < 0.05$) between antiviral efficacy distribution of 75 mg, 150, 300 and 450 mg.

	EC_{50}^S (μ M)		
Dose (mg)	0.5	10	35
75	0.48*	0.22*	0.08*
150	0.48	0.31*	0.15*
300	0.49*	0.39*	0.23*
450	0.50*	0.43*	0.29*

* Statistically significant

Table S2. Comparison of antibacterial efficacy median values for different EC_{50}^S values and different dose regimens with intake frequency of twice per day and treatment duration of 5 days. We indicate the antibacterial efficacy distribution showing statistical significance ($P < 0.05$).

	EC_{50}^S (μ M)		
Dose (mg)	0.5	10	35
75	0.10*	0.009*	0.003*
150	0.16*	0.015*	0.006*
300	0.23*	0.034*	0.01*
450	0.44*	0.042*	0.02*

* Statistically significant

Table S3. Comparison of antiviral and antibacterial efficacy median values for different drug efficacy EC_{50}^{S} and dose regimen of 75 mg with different intake frequency and treatment duration of 5 days. We denote treatments with statistical significance ($P < 0.05$).

Intake frequency	0.5	EC_{50}^{S} (μ M) 10	35
		**Antiviral efficacy**	
twice per day	0.48*	0.22*	0.08*
one per day	0.44*	0.13*	0.05*
		**Antibacterial efficacy**	
twice per day	0.10*	0.009*	0.003*
one per day	0.04*	0.003*	0.001*

* Statistically significant

Table S4. Comparison of antiviral and antibacterial efficacy median values for different drug efficacy EC_{50}^{S} with intake frequency of twice per day and different treatment duration. There is no statistical significance ($P > 0.05$) between antiviral/antibacterial efficacy distributions obtained with different treatment durations.

Treatment duration	0.5	EC_{50}^{S} (μ M) 10	35
		**Antiviral efficacy**	
5 days	0.48	0.22	0.08
10 days	0.54	0.25	0.10
		**Antibacterial efficacy**	
5 days	0.10	0.009	0.003
10 days	0.12	0.009	0.003

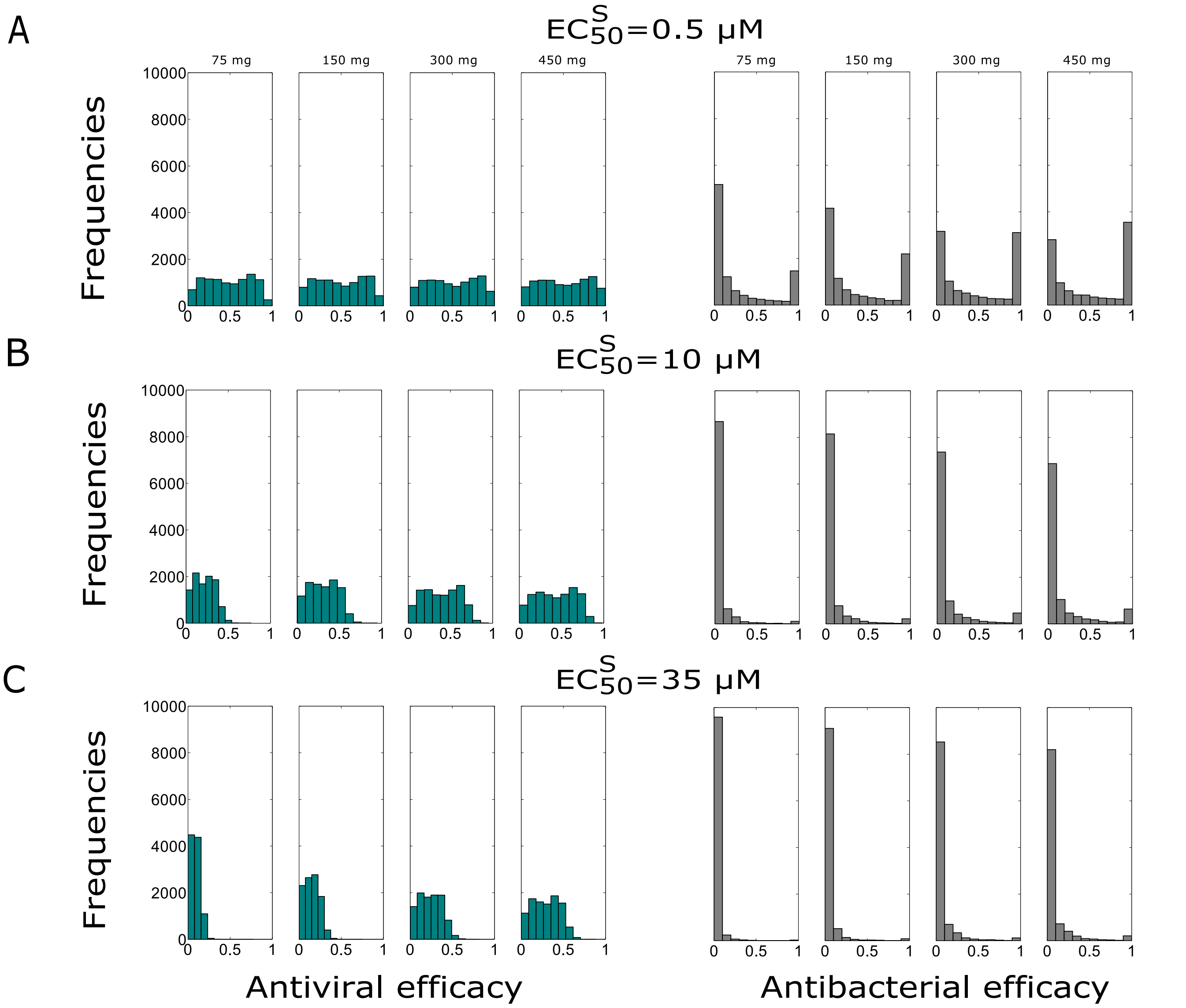

Supplementary Figure S1. Histograms of antiviral (green) and antibacterial (gray) efficacy for dose of 75, 150, 300 and 450 mg, intake frequency of twice per day and treatment duration of 5 days. This is tested for (A) $EC^S_{50} = 0.5\ \mu M$, (B) $EC^S_{50} = 10\ \mu M$, (C) $EC^S_{50} = 35\ \mu M$. The x-axis represents the Oseltamivir antiviral efficacy against IAV ranging from 0 (0%) to 1 (100%).

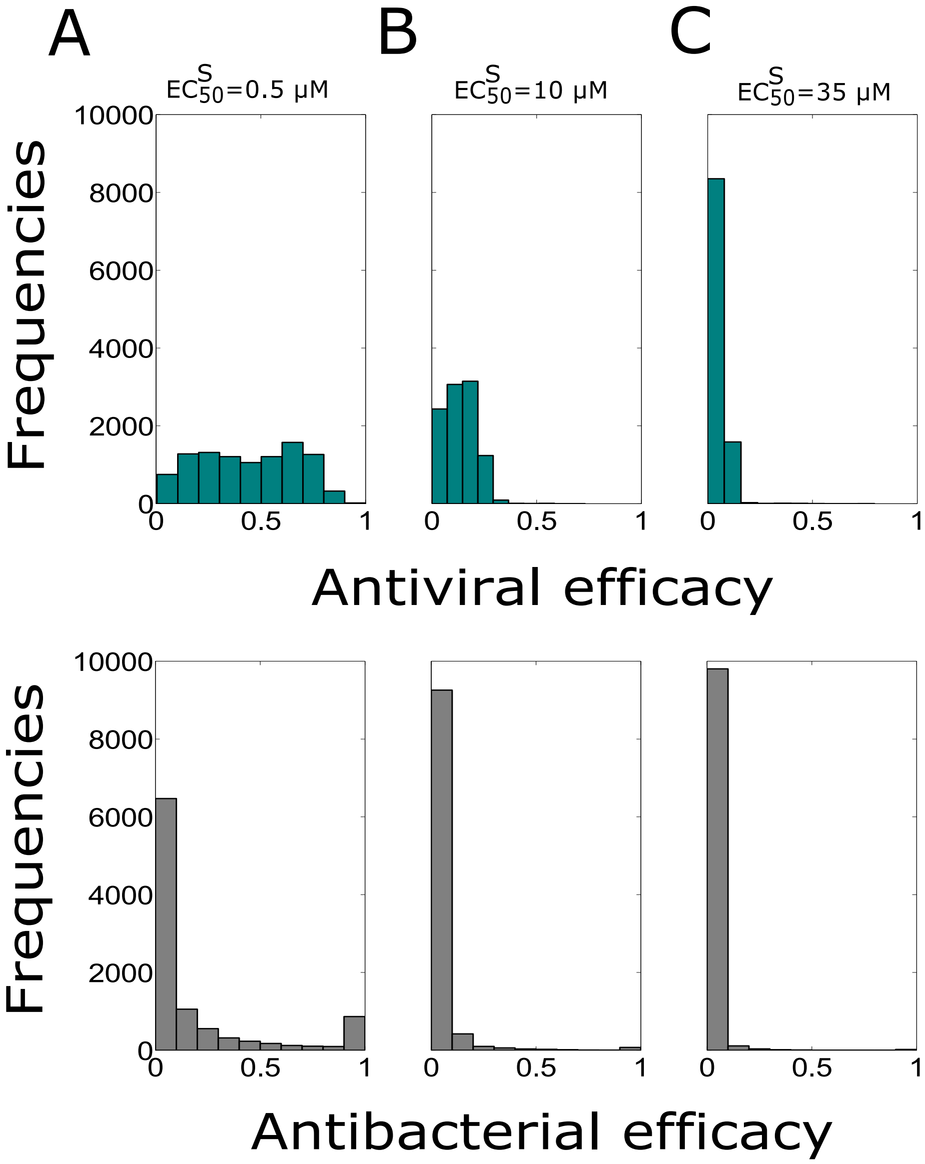

Supplementary Figure S2. Histograms of antiviral (green) and antibacterial (gray) efficacy for dose of 75 mg, intake frequency of one per day and treatment duration of 5 days. This is tested for (A) $EC_{50}^{S} = 0.5$ μM, (B) $EC_{50}^{S} = 10\ \mu M$, (C) $EC_{50}^{S} = 35\ \mu M$. The x-axis represents the Oseltamivir antiviral/antibacterial efficacy against IAV/Sp ranging from 0 (0%) to 1 (100%).

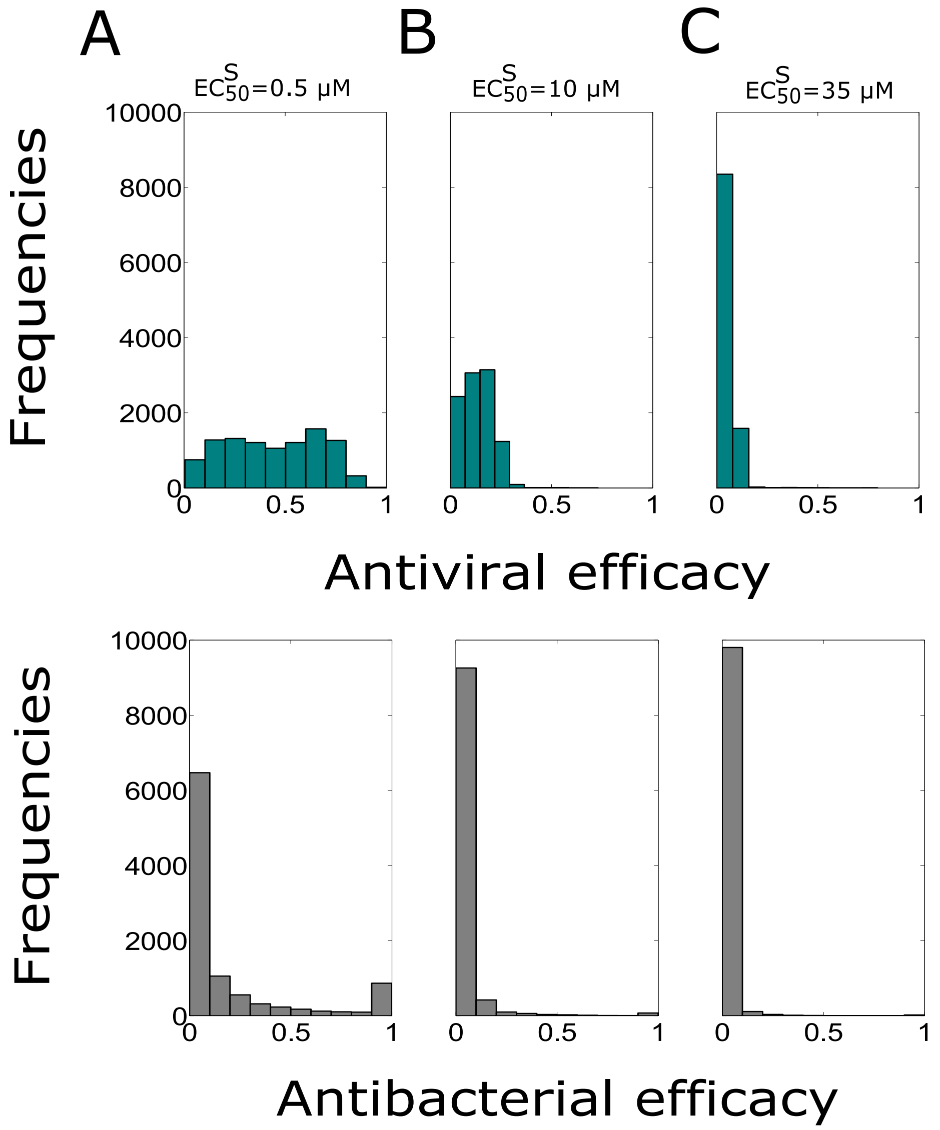

Supplementary Figure S3. Histograms of antiviral (green) and antibacterial (gray) efficacy for dose of 75 mg, intake frequency of twice per day and treatment duration of 10 days. This is tested for (A) $EC_{50}^{S} = 0.5\ \mu M$, (B) $EC_{50}^{S} = 10\ \mu M$, (C) $EC_{50}^{S} = 35\ \mu M$. The x-axis represents the Oseltamivir antiviral/antibacterial efficacy against IAV/Sp ranging from 0 (0%) to 1 (100%).

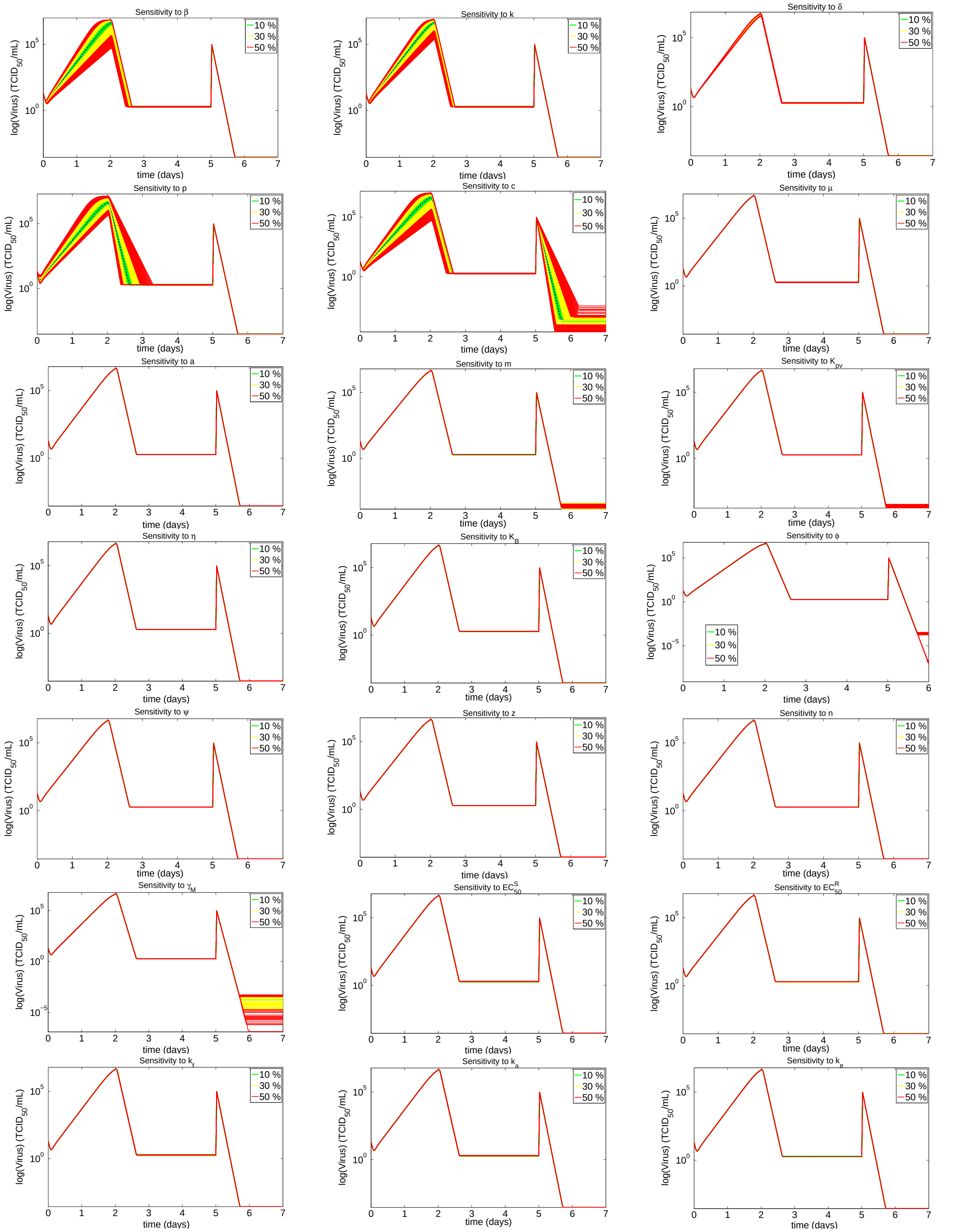

Supplementary Figure S4. IAV dynamics derived from the sensitivity analysis, varying each model parameter once per time of 10% (green), 30% (yellow) and 50% (red) respectively. The blue dashed line represents the viral titers obtained with nominal parameters. We used the treatment with 75 mg of dose, twice per day for 5 days. The initial condition for IAV is $V(0) = 20$ $\text{TCID}_{50}\text{mL}^{-1}$.

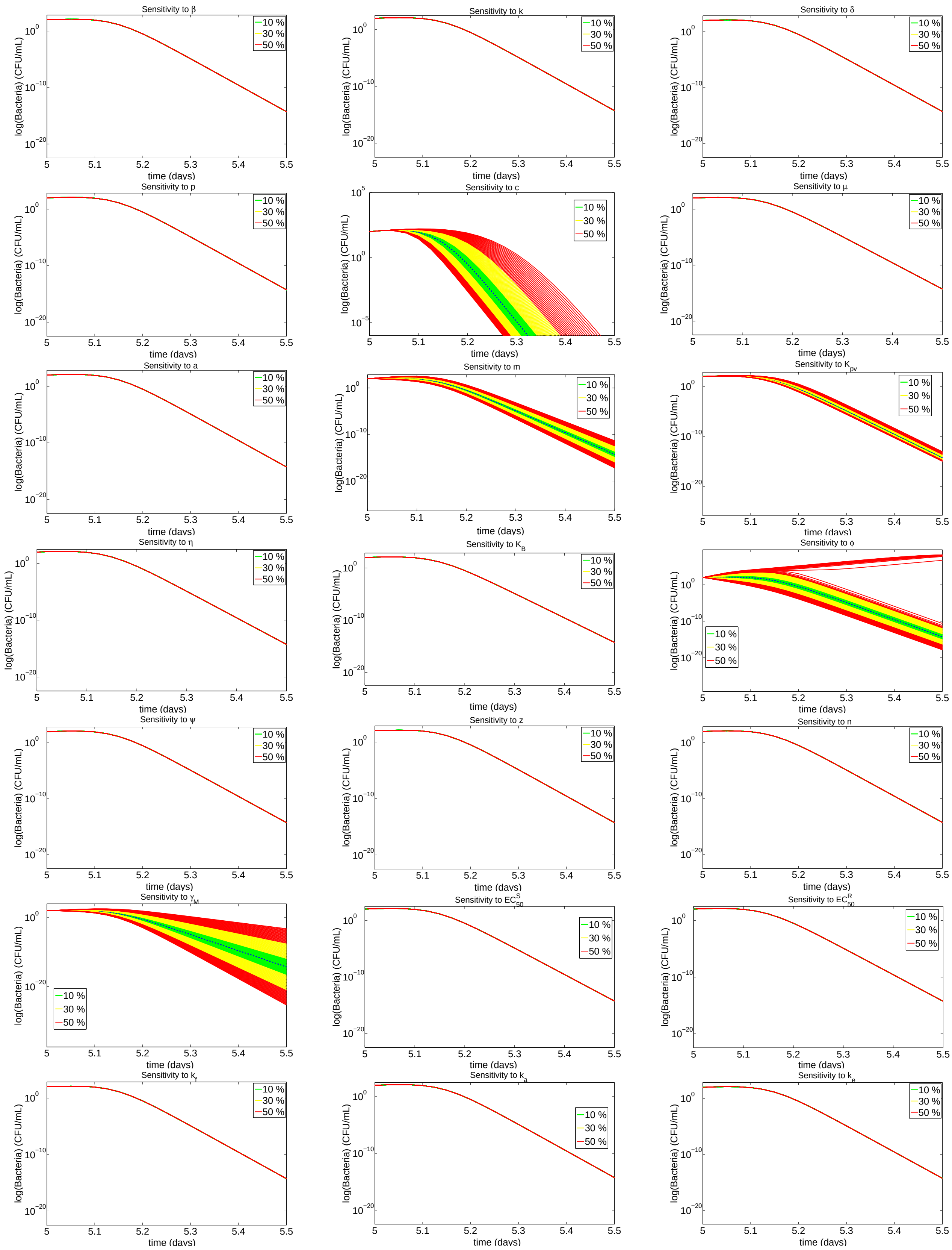

Supplementary Figure S5. Bacterial concentration from the sensitivity analysis, varying each model parameter once per time of 10% (green), 30% (yellow) and 50% (red). The blue dashed line represents the bacterial concentration obtained with nominal parameters. We used the treatment with 75 mg of dose, twice per day for 5 days. The initial condition for the bacteria is $B(0) = 100$ CFUmL^{-1}.
